# Prevalence and correlates of metabolic syndrome among women living in urban slums, Mysore, India

**DOI:** 10.1371/journal.pgph.0000846

**Published:** 2023-07-07

**Authors:** Karl Krupp, Arathi P. Rao, Benjamin Pope, Kavitha Ravi, Anisa Khan, Vijaya Srinivas, Purnima Madhivanan, Arun Srinivas

**Affiliations:** 1 Division of Public Health Practice & Translational Research, Mel & Enid Zuckerman College of Public Health, University of Arizona, Tucson, United States of America; 2 Public Health Research Institute of India, Mysore, India; 3 Prasanna School of Public Health, Manipal Academy of Higher Education, Manipal, India; 4 Department of Epidemiology & Biostatistics, Mel & Enid Zuckerman College of Public Health, University of Arizona, Tucson, Arizona, United States of America; 5 Department of Health Promotion Sciences, Mel & Enid Zuckerman College of Public Health, University of Arizona, Arizona, Tucson, United States of America; 6 Division of Infectious Diseases, College of Medicine, University of Arizona, Tucson, Arizona, United States of America; 7 Department of Cardiology, Apollo Hospital, Mysore, India; University of South Carolina Arnold School of Public Health, UNITED STATES

## Abstract

Metabolic Syndrome (MetSyn) is a predictor of cardiovascular disease (CVD). About a third of urban Indians suffer from MetSyn. This study examined the prevalence of MetSyn among women living in urban slums. A cross-sectional survey was carried out between October 2017 and May 2018 among a non-probability sample of slum-dwelling women, 40–64 years of age, in six government-designated slums in Mysore, India. Data were collected on demographics, diet, behavioral risks, anthropometry, blood pressure, serum glucose, hemoglobin A1c, and serum lipids. The study used a definition of MetSyn from the International Diabetes Federation Task Force on Epidemiology and Prevention with an HbA1c measure for average blood glucose. About two-fifths of the 607 participants had MetSyn (41.5%; 95% CI: 37.7–45.5). Of those, 40.9% met three criteria, 38.1% four, and 25.0%, all five criteria. Elevated BP was the most prevalent MetSyn factor (79.6%), followed by increased waist circumference (54.5%), low HDL (50.1%), elevated Hb A1c (37.1%), and elevated triglycerides (36.1%). Odds for MetSyn were 1.52 times greater for those who were 50–59 years of age compared with those 40–49 years of age (adjusted odds ratio[AOR]:1.52; 95% CI:0.96–2.40). Women with mobility issues had 1.29 times higher odds of MetSyn than those without it (AOR: 0.76, 95% CI: 0.96, 1.75). Housewives had 1.29 times greater odds of MetSyn (AOR: 1.29, 95% CI: 1.00, 1.67). There is a high prevalence of MetSyn among urban slum-dwelling women in Mysore. There is a need for interventions aimed at reducing CVD risk factors in this population.

## Introduction

Metabolic Syndrome (MetSyn), a cluster of metabolic abnormalities elevating risk for Diabetes Type 2 (T2DM) and Cardiovascular disease (CVD), affects about one-in-four adults globally [1]. Individuals with MetSyn have a two to three-fold increased risk of developing CVD, and about a fivefold increased risk for incident T2DM [2]. While MetSyn prevalence is highest in developed nations, it is rapidly increasing in low-and-middle-income countries (LMIC). Some of the highest LMIC rates are found in India where the largest increases have been among middle-aged urban females [3].

While there is controversy around the pathophysiology and clinical value of Metsyn [4], there is growing evidence of its usefulness in public health interventions and health system planning [5, 6]. Studies and clinical trials have shown population level effectiveness for nutritional and lifestyle interventions addressing Metsyn in both developed and developing countries [7–10]. Research demonstrates the importance of identifying populations with Metsyn since without intervention, they utilize higher levels of inpatient, primary care, and other outpatient and pharmacy services compared to those without metabolic disorders [11]. A study examining healthcare costs for elderly patients with Metsyn found that overall costs were approximately 20% higher compared to costs for patients without the syndrome [12]. Currently India spends approximately one quarter of its healthcare expenditures on chronic diseases and that percentage continues to grow [13]. It is estimated that the burden of metabolic-related disorders will cost India $6.2 trillion between 2012–2030, and contribute to $4.8 trillion in lost economic output by 2030 [14], making prevention a public health priority.

A number of studies have examined the prevalence of MetSyn among community samples in different regions of India, but little is actually known about rates for most parts of the country. Between 2010 and 2019, only 11 studies were identified for six of India’s 29 states, and two of the country’s eight Union Territories. The Northern region appeared to have generally higher combined rates with urban Chandigarh (35.8%) the highest, followed by an urban sample in Jammu and Kashmir (35.2%), and rural samples in Uttarakhand (28.6%) and Uttar Pradesh (11.7%); the first three using Adult Treatment Panel III (ATP-3), and the fourth, a modified clinical criterion, respectively [15–18]. In Western India, two studies in urban Mumbai had combined rates of 19.5% and 40% (ATP-3), and a rural sample Maharashtra, a combined 5% prevalence (ATP-3) [19–21]. In Eastern India, an urban Orissa sample had a 33.5% combined MetSyn prevalence (ATP-3), and a study in rural West Bengal a 15.4% combined prevalence (ATP-3 modified criteria) [22]; while in Southern India, studies in Kerala [23] and rural Puducherry Union Territory [24] reported rates of 24% and 39.7% with ATP-3 and the International Diabetes Federation (IDF) criteria, respectively. Finally, a study in rural Karnataka found a combined prevalence of 25.9% (ATP-3) [25]. In all studies reporting disaggregated data, females had markedly higher MetSyn rates compared with males. In Berhampur City, Orrisa for instance, 42.3% of women had MetSyn, a rate 69.5% higher than for men (24.9%) [26]. In other studies, prevalence for women was 90% higher compared to men in West Bengal [22], 44% higher in Uttar Pradesh [27], 40% higher in Kerala [23], and 6% higher in Puducherry [24]. Across most studies, elevated body mass index (BMI), dyslipidemia, and insulin resistance contributed disproportionately to the prevalence of MetSyn in women, while in men, hypertension and hypertriglyceridemia were relatively more important.

According to the census, approximately 64 million Indians live in urban slums. This represents about 17.4% of all city households and one-third of India’s 1.2 billion people [28]. Only two studies were found examining the prevalence of Metsyn among Indian urban slum dwellers. A 2012 study among 350 adults in urban slums in Hyderabad, India, found a prevalence of 17.1% in men and 29.4% in women [29]. Another study of 169 residents of a slum in Pune, India, found Metsyn was associated with higher age (53%), being a housewife (36.7%), having a secondary education (36.7%), and being part of a lower socioeconomic lower class (36%) [30]. Given the large population in Indian urban slums—and substantial evidence that Metsyn is associated with urban residence [31], economic and social vulnerability, and the high documented rates of diabetes, obesity, hypertension, and smoking in Indian slums [32–34]—there is a need for more research examining the prevalence of Metsyn in India’s slum populations,. Data informing public health interventions for metabolic disease in India’s most vulnerable populations is critically needed to avoid higher health and welfare expenditures that inevitably reduce national productivity and competitiveness [35].

Currently there are large disparities in the burden of reported non-communicable diseases among women compared with men, in India [36]. A study of 699,686 women and 103,525 men aged 15–49 from the Government of India Demographic and Health Surveys, 2015–16, found that in South India, women were almost twice as likely to report at least one noncommunicable disease (NCD), at 95 per 1000, compared to men at 54 per 1000 [36]. Middle-aged women had the highest rate of reporting one NCD (Odds Ratio [OR] 4.07; 95% Confidence Interval [CI] 3.91, 4.24), 11% higher than the same age category among men (OR: 3.67; CI: 3.17,4.25). More women were also likely (13.5 per 1000) than men (10.2 per 1000) to report a heart-disease-related NCD [36] and NCD rates are increasing faster among women than men [37]. Finally, in high income countries, present presentation with an NCD typically comes at age 55 years or older, while in India, onset occurs earlier at 45 years or less, making NCD screening at earlier ages critical for NCD control and treatment. For all these reasons, the objective of this study was to assess the burden of Metsyn, an early indicator of NCD risk, among 600 middle-aged, urban-slum-dwelling Indian women, ages 40 to 64 years of age.

## Materials & methods

Between October 2017 and May 2018, a cross-sectional study was conducted to examine the prevalence of MetSyn and its components in a convenience sample of 607 women living in six government-designated slums in Mysore, India. We selected nonprobability sample because of the difficulty of using population-based sampling in slums with few or no official addresses, high mobility, and poorly defined community borders. According to the 2011 Census, Mysore had a population of 893,062, of which 446,386 were females. About 19% of the city’s population lived below the poverty line, and approximately 49,352 residents residing in slums.

### Ethics statement

A protocol for the study was approved by Institutional Review Boards at University of Arizona, USA, and Public Health Research Institute of India, Mysuru, India. All participants provided written informed consent prior to data collection.

### Sample size

The study sample size was calculated based on an estimated 39,029 slum-dweller population in Mysore [38], a 5% precision, 99% confidence level, and a 30% estimated prevalence of MetSyn. This resulted in a sample size of 552 which we increased to 600 to account for nonresponse and incomplete data. We randomly selected six communities for the study from a sampling frame of 63 communities officially designated as "notified" slums by the Karnataka Slum Development Board.

### Study recruitment

Study staff visited the designated slums one day prior to recruitment and distributed brochures describing the purpose of the study, eligibility criteria, and study activities. Women who expressed interest in the study were screened based on the inclusion and exclusion criteria. To be included, participants had to be female; aged 40–64 years (This age range was selected because the median age for presentation with a first myocardial infarction among women in India is 58 years, and we hoped to assess women in age groups where lifestyle intervention would have the most benefits in preventing cardiovascular disease); residents of urban Mysore (defined as living in Mysore City for a period of six months or more); and willing and able to undergo informed consent and ability to complete all study procedures including a medical examination, venous blood draw, a resting electrocardiogram (ECG), and an interviewer-administered survey. Exclusion criteria included having a known history of hemophilia, hospitalization within the past three months, and having any condition that might pose a risk for undergoing study procedures. A brief anonymous survey was used to collect demographic information from women who declined enrollment in order to assess any systematic biases in participant recruitment. Prior to data collection, potential participants underwent an informed consent process. Study staff explained the study purpose, read the informed consent document verbatim, described all study procedures, and answered any questions from potential participants.

### Data collection and measures

Data were collected using an interviewer-administered questionnaire adapted from the Cardiometabolic Risk Reduction in South Asia (CARRS) Surveillance Study [39]. The study questionnaire was translated into the local language of *Kannada* and validated using methods described elsewhere [40]. The adapted instrument was pretested in a sample of 28 women recruited from Mysore slums and their input informed the final version used in the study.

### Medical procedures

Anthropometry measurements were carried out by trained staff. Measurements were taken three times and a mean measurement recorded. Height was measured without shoes using an IndoSurgicals Stadiometer Height-Rod model 20015. Weight was measured on a calibrated Hoffen Digital Electronic LCD Weighing Scale model BDCC-H017 to the nearest 100 grams. Waist circumference was measured using an ADC woven measuring tape at the midpoint between the lower border of the rib cage and upper border of the iliac crest. Blood pressure (BP) was measured using a Bpl B3 120/80 Blood Pressure Monitor in sitting position with readings taken three times, and the average systolic and diastolic blood pressure recorded as recommended by the American Heart Association.

### Biochemistry and electrocardiography

All biomedical samples were collected onsite by trained phlebotomist at Public Health Research Institute of India diagnostic laboratory in Mysore City. Venous blood was collected for HbA1c testing and lipid profile and tested the same day. Samples were centrifuged at 3000 RPM in a microcentrifuge for five minutes. Serum was tested for glycated hemoglobin (HbA1c), triglycerides (TG), total cholesterol (TC), low-density lipoproteins (LDL), and high-density lipoproteins (HDL) using a BioSystems BTS-350 semi-automatic analyzer. Prior to use, the system was calibrated and verified using pretested samples. A random set of samples were simultaneously sent to an external accredited laboratory for quality control. ECGs were performed onsite by a trained medical technician using a BPL CARDIART 9108 three channel ECG (BPL Medical Technologies PVT LTD, Bangalore, India) with 12-lead simultaneous acquisition and Arrhythmia Detection in auto mode. Prior to data collection, the ECG machine was calibrated according to manufacture specification. ECGs were electronically transferred to a Cardiologist at the Cardiology Department at Apollo Hospital, Mysore, for reading and interpretation.

### Primary outcome

The primary outcome of this study was the presence of metabolic syndrome (MetSyn) as defined by the harmonized definition [41] using HBA1c as a measure of average serum glucose and an HbA1c ≥5.7% cutoff suggested by the American Diabetes Association [42]. Women meeting at least three of the following criteria were classified as having MetSyn if they had:

- A waist circumference ≥80 cm
- Elevated triglycerides (TG) (≥150 milligrams per deciliter [mg/dL])
- HDL cholesterol levels < 50 mg/dL
- Elevated blood pressure (≥130/85 mmHg) or on drug treatment for hypertension
- Elevated HbA1c (HbA1c ≥5.7%) or drug treatment for diabetes mellitus.

### Explanatory variables

Twenty explanatory variables with the potential to influence the presence of MetSyn were selected on the basis of a literature review: age; education; religion; marital status; work status; annual household income; household size; current use of tobacco products; current use of smokeless tobacco products; alcohol use; frequency of adding salt/having salted pickles; frequency of adding salt/salty seasoning when cooking food; frequency of consuming processed food high in salt; type of oil/fat used for meal preparation; consumption of sweetened beverages; consumption of meat products; level of physical activity; sedentary behavior; and history of heart attack/chest pain from heart disease/stroke. Education was defined as *no formal education*, *primary school or less*, and *secondary school or highe*r. Religion was defined as *Hindu* and *Other*, which included Muslim and Christian. Caste was defined as *lower* and *higher*, with lower including scheduled tribes, scheduled castes, and other lower castes; and higher including general castes. Marital status was defined as *married; never married*; and *Other*, which included widowed and separated. Work status was defined as *self-employed*; *housewife*; and *Other*, which included government employees, nongovernment/private employees, retired people, and unemployed people able to work. Annual household income was estimated using self-reported *annual*, *monthly*, or *weekly household income*. For participants who did not report an annual household income, annual household income was calculated by multiplying monthly household income (if reported) by 12 months/year or weekly household income (if reported) by 52.143 weeks/year. The estimated annual household income was then converted from Indian Rupees (INR) to United States dollars (USD) using an approximation of the conversion rate at the time that the survey was administered (i.e., the average of the conversion rates for December 31, 2015 and December 31, 2016 [1 USD = 67.06 INR] (World Currency Exchange Rates and Currency Exchange Rate History, 2017). Physical activity level was measured based on self-reported hours, with each respondent being assigned to the category (*sedentary/mild/moderate*) for which they reported the greatest number of hours.

### Statistical analyses

Data were presented as frequencies and percentages for categorical variables, and as mean (standard deviation [SD]) and median (first quartile [Q1], third quartile [Q3]) for continuous variables. Differences in socio-demographic, health behavior, and medical history variables by MetSyn were assessed using chi-squared (χ^2^) tests and analysis of variance (ANOVA) for categorical and continuous variables, respectively. Analyses were repeated to assess univariate effects as well as adjusting for covariates. Additionally, as participants within a given slum were potentially correlated, adjustment for clustering was performed by using the Mantel-Haenszel test and simple linear and logistic regression. Logistic regression analyses were conducted to identify variables associated with MetSyn. Variables found to be conservatively associated with MetSyn using χ^2^ test, ANOVA or cluster-adjusted linear regression (p<0.20) or those of clinical importance were selected *a priori* to be included in the model. Variables were excluded from the model if there was little variation in response (i.e., if ≥90% of the sample fell into a single response category), or if variables were highly correlated. Correlation and multicollinearity were assessed using Pearson’s correlation coefficients and variance inflation factors (VIFs), respectively [43]. Each model was run twice: once adjusting for clustering and the other assuming no clustering. All analyses used a two-tailed significance level of α ≤ 0.05 and were performed using Stata version 11.2 (StataCorp, College Station, TX).

## Results

### Characteristics of the sample

The mean age of the 607 women was 50.0 years, and 59.5% had no formal education (Table 1). About 61.5% of respondents worked in some occupation other than reporting to be a housewife, 13.5% reported using smokeless tobacco products, 53.9% reported being mildly or moderately active, 61.8% reported some mobility issues, 8.9% reported currently consuming alcohol; 48.8% ate salted pickles/salty pickled foods at least once a week; and 23.4% reported taking prescribed allopathic medication for hypertension. Additional variables are listed in Tables 1 and 2. There were a total of seven “clusters”, with a median size of 74 participants, and a mean of 86.7 participants. The intra- class correlation coefficient (ICC) was 0.033.

**Table 1:** Sociodemographic characteristics of the study population from Mysore, India (N = 607).

Characteristic	n (%)
Age in years	
Mean (SD)	50·0 (7.32)
Median (Q1, Q3)	50 (44, 55)
Highest level of education	
No formal education	361 (59·5)
Primary school or less	179 (29·5)
Secondary school or higher	67 (11·0)
Religion	
Hindu	511 (84·2)
Other	89 (15·8)
Marital status	
Married	312 (51·4)
Other (Never married, widowed, separated)	295 (48·6)
Work status	
Employed	373 (61·5)
Housewife	234 (38·6)
Monthly household income (INR)	
< 3000	129 (21·3)
3000–10000	321 (53·0)
10001–20000	119 (19·6)
> 20000	37 (6·2)
Smokeless tobacco use	
No	525 (82.5)
Yes	82 (13.5)
Physical activity level	
Sedentary	280 (46.1)
Mild	175 (28.8)
Moderate	152 (25.0)
Mobility issues	
No	232 (38.2)
Yes	375 (61.8)
Current alcohol use	
No	54 (8.9)
Yes	553 (91.1)
Frequency of eating pickles or pickled food	
Never	242 (39.9)
Monthly	69 (11·4)
Weekly	150 (24.7)
Daily	146 (24·1)
Taking medication for hypertension	
No	465 (76.6)
Yes	142 (23.4)

Frequencies and percentages were presented for categorical variables and mean (standard deviation [SD]) and median (first quartile [Q1], third quartile [Q3]) for continuous variables. SD = standard deviation; Q1 = first quartile; Q3 = third quartile; INR = Indian rupee.

**Table 2:** Factors associated with metabolic syndrome among women living in urban slums in Mysore, India.

Characteristic	Metabolic syndrome			
	Yes	No	p- value	Clustering p-value
	n (%) (n = 252)	n (%) (n = 355)		
**Socio-demographics**				
Mean age in years (SD)	50·4 (7·23)	49·8 (7·38)	0·31	0·52
Religion				
Hindu	201 (79·8)	310 (87·3)	***0·01***	0·10
Other	51 (20·2)	45 (12·7)		
Highest level of education				
No formal education	137 (54·4)	224 (63·1)	***0·02***	0·04
Primary school or less	81 (32·1)	98 (27·6)		
Secondary school or higher	34 (13·5)	33 (9·3)		
Marital status				
Married	139 (55·2)	173 (48·7)	0·12	0·17
Other	113 (44·8)	182 (51·3)		
Work status				
Employed	144 (57·1)	229 (64·5)	0·07	0·03
Housewife	108 (42·9)	126 (35·5)		
(Monthly) household income (INR)				
< 3000	58 (23·0)	71 (20·0)	0·32	0·28
3000–10000	134 (53·2)	187 (52·8)		
10001–20000	45 (17·9)	74 (20·9)		
>20000	15 (6·0)	22 (6·2)		
**Health behaviors**				
Currently use smokeless tobacco products				
Yes	29 (11·5)	53 (14·9)	0·22	0·26
No	223 (88·5)	302 (85·1)		
Currently consume alcohol				
Yes	16 (6·3)	38 (10·7)	0·06	0·33
No	236 (93·7)	317 (89·3)		
Frequency of eating pickles or pickled food				
Never	110 (43·7)	132 (37·2)	0·14	0·18
Monthly	29 (11·5)	40 (11·3)		
Weekly	55 (21·8)	95 (26·8)		
Daily	58 (23·0)	88 (24·8)		
Consuming cold beverages (sodas, colas etc)				
Never	175 (69·4)	231 (65·1)	0·28	0·36
Monthly	46 (18·3)	73 (20·6)		
Weekly	31 (12·3)	51 (14·4)		
Consuming non-seasonal fruit			0.49	0.6
No	51 (20.2)	64 (18.0)		
Yes	201 (79.8)	291 (82.0)		
Consuming western sweets			0.33	0.52
No	190 (75.4)	255 (71.8)		
Yes	62 (24.6)	100 (28.2)		
Mobility issues			0.22	0.09
No	143 (40.3)	89 (35.3)		
Yes	212 (59.7)	163 (64.7)		
Level of physical activity				
Sedentary	118 (46·8)	162 (45·6)	0·70	0·96
Mild	73 (29·0)	102 (28·7)		
Moderate	61 (24·2)	91 (25·6)		
Medical history				
Reported chest pain				
Yes	66 (26·2)	118 (33·2)	0·06	0·02
No	186 (74·8)	237 (66·8)		

For binary and continuous variables, logistic regression was used to assess the association between the variable and presence of absence of MetSyn; for ordinal variables, Mantel-Haenszel’s test for trend was utilized. SD = standard deviation; INR = Indian rupee

### Prevalence of MetSyn

**Fig 1:**
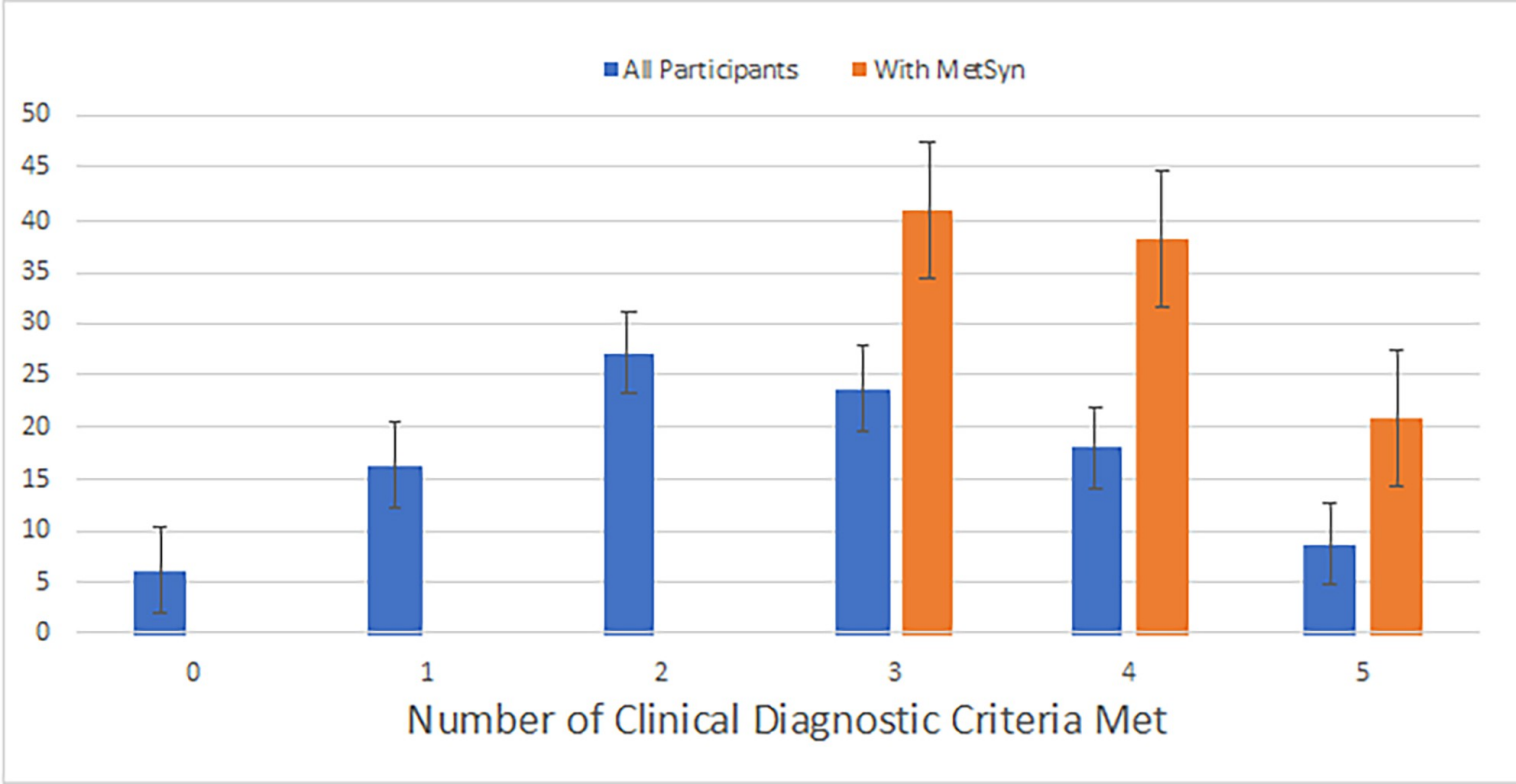
Number of women with metabolic syndrome conditions in urban Mysore, India.

**Fig 2:**
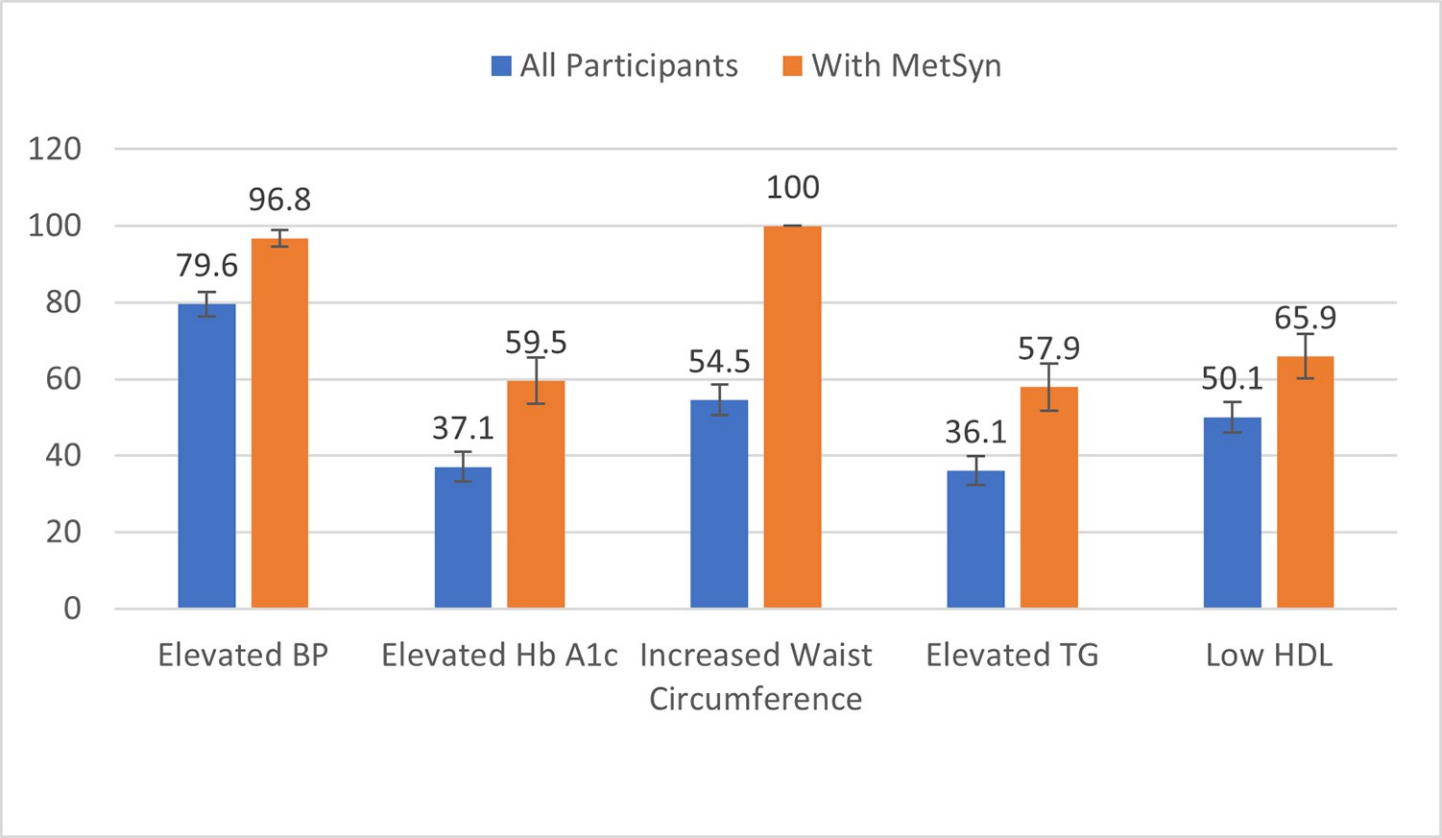
Clinical diagnostic criteria among all participants and only those with metabolic syndrome in urban slums in Mysore, India.

The overall percentage of women with MetSyn was 41.5% (n = 252; 95% CI: 0.38–0.46). Of those, 40.9% (95% CI: 0.35–0.48) met three of five criteria; 38.1% (95% CI: 0.32–0.45), four criteria; and 21% (95% CI: 0.15–0.28), all five criteria. (Fig 1) Among the entire sample, elevated BP was the most prevalent criteria (79.6%), followed by increased waist circumference (54.5%), low HDL (50.1%), elevated HbA1c (37.1%), and elevated triglycerides (36.1%). (Fig 2). Among the criteria for MetSyn and demographic variables, the proportions with high glucose, high triglycerides, and low HDL were significantly higher in those who met all five criteria versus those who only met three or four. Additionally, the proportion of women who reported being housewives was significantly higher among those who met only three or four of the criteria.

### Characteristics of women with MetSyn

Women with MetSyn were significantly (45.6%) more likely to have a formal education compared to participants without MetSyn (36.9%) (p = 0.02) (Table 2). A greater proportion were housewives (p = 0.07) and non- alcohol consumers (p = 0.06), although only marginally significantly so. Additional differences between those with MetSyn and those without which were not significant included: being older (p = 0.31), being more sedentary (p = 0.70), eating non-seasonal fruits (p = 0.49), not eating Western-style sweets (p = 0.33); having mobility issues (p = 0.22); having higher parity (p = 0.46) and reporting more pregnancies (p = 0.41).

### Variable selection for logistic regression model

The following factors were included in the logistic regression model: age (when treated as categorical); highest level of education; employment status; consumption of seasonal fruits; mobility issues; frequency of eating salted pickles/salty pickled foods; consumption of sodas; religion; and history of protracted chest pain (used as a proxy for history of heart attack or chest pain from heart disease or stroke). Alcohol use was excluded with 90% of respondents not currently drinking alcohol, as were eating legumes and pulses, eating cooked vegetables, and eating leafy greens. Although when considered as a continuous variable, age was not associated with MetSyn, however, when collapsed into the categories 40–49 years, 50–59 years, and 64+ years, it was conservatively associated with MetSyn. The following factors were also excluded because they were not conservatively associated with MetSyn (p>0.25): meat consumption; parity; number of pregnancies; caste; education; currently using smokeless tobacco; physical activity; eating any fruit; drinking fruit juice; eating raw vegetables, consuming sodas, eating Indian deep-fried foods, eating Western deep-fried foods; eating Indian sweets; and eating Western sweets. In the multiple logistic regression model adjusting for clustering, the VIF for marital status exceeded four, so it was removed.

### Factors associated with MetSyn

Results of the logistic regression analyses are presented in Table 3. Being aged 50–59 was marginally associated with MetSyn in the multiple regression model as was mobility issues. Being a housewife and experiencing chest pain were significantly associated with MetSyn, eating seasonal fruits, and religion were not associated with MetSyn in the multiple regression model. Odds of MetSyn were 1.52 times higher for those 50–59 years of age, as compared with those 40–49 years of age (adjusted odds ratio [AOR]:1.52; 95% CI:0.96–2.40). Women who were housewives had 1.14 times greater odds of MetSyn (AOR: 1.14, 95% CI: 1.00–1.29) compared to those who were employed. Women who had chest pain had 0.68 times lower odds of MetSyn (AOR: 0.68, 95% CI: 0.51, 0.91) than those who did not. Those who had mobility issues had 1.29 times greater odds of MetSyn (AOR: 1.29, 95% CI: 0.96, 1.75) than those who did not.

**Table 3:** Odds of having metabolic syndrome among women living in urban slums in Mysore, India with adjustment for clustering.

Characteristic	Metabolic Syndrome			
	Unadj OR	95% CI	Adj OR	95% CI
Age in categories (in years)				
40–49	Ref.		Ref.	
50–59	1·59	(0·93, 2·72)	1·52	(0·96, 2·40)
60–64	1·15	(0·66, 2·00)	1·05	(0·67, 1·63)
Highest level of education				
No formal education	Ref.			
Primary school	1·35	(0·78, 2·35)		
Secondary school or higher	1·68	(0·65, 4·35)		
Marital Status				
Single/Widowed/Separated	Ref.			
Married	0·77	(0·53, 1·12)		
Current use of smokeless tobacco				
No	Ref.			
Yes	0·74	(0·44, 1·25)		
Physical activity level				
Sedentary	Ref			
Mild	0·98	(0·68, 1.41)		
Moderate	0·92	(0·78, 1·09)		
Employment status				
Housewife	**1·17**	**(1·02, 1·35)**	**1·14**	**(1·00, 1·29)**
Employed	Ref.		Ref.	
Seasonal fruits				
No	Ref.		Ref.	
Yes	**0·70**	**(0·50, 0·98)**	0·75	(0·51, 1·10)
Consuming Western Sweets				
No	Ref.			
Yes	0·83	(0·48, 1·45)		
Income				
<3000	Ref.			
3000–10000	0·88	(0·44, 1·74)		
10001–20000	0·74	(0·48, 1·17)		
>20000	0·83	(0·58, 1·21)		
Chest pain				
No	Ref.		Ref.	
Yes	**0·71**	**(0·54, 0·95)**	**0·68**	**(0·51, 0·91)**
Frequency of eating pickles or pickled food				
Never	Ref.			
Monthly	0· 87	(0·59, 1·27)		
Weekly	0·69	(0·40, 1·21)		
Daily	0·79	(0·52, 1·21)		
Sodas				
Never	Ref.			
Monthly	0·83	(0·62, 1·12)		
Weekly	0·80	(0·47, 1·37)		
Religion				
Hindu	Ref.		Ref.	
Other	1·75	(0·90, 3·41)	1·79	(0·89, 3·60)
Mobility Issues				
No	Ref.		Ref.	
Yes	1·24	(0·97, 1·58)	1·29	(0·96, 1·75)
Parity (# of live births)	1·04	(0·96, 1·13)		
Number of pregnancies	1·03	(0·96, 1·10)		
Current alcohol use				
No	Ref.			
Yes	0·57	(0·18, 1·77)		

AOR: Adjusted Odds Ratio. AORs were obtained using simple logistic regression (left two columns) and multiple logistic regression (right two columns).

## Discussion

This study is the first we are aware of that examined the prevalence of MetSyn in Indian slum-dwelling women, and found about 40% of them had MetSyn. Of the five IDF criteria, elevated BP was the most pervasive in this population, with four-fifths of participants having hypertension. Over half of women had a waist circumference ≥80 cm, and a little less than half reported being sedentary. Three in five women reported working outside of the home, and this was found to be protective against MetSyn. Other studies have shown that MetSyn predicts mobility decline in the elderly since comorbid obesity and T2DM are associated with recurrent falls, diabetic foot ulcers, and myalgia; all of which predict declining mobility in the elderly [44]. Finally, the prevalence of MetSyn at 41.5%, in a sample of slum-dwelling women, is alarmingly high. Research in other urban female samples for instance, have previously reported MetSyn prevalence ranging from 12.6% [19] to 29.6% [30] in similar populations. This may presage an epidemic of stroke and heart disease and associated health system costs. Public health authorities should prioritize screening and treatment of the most common MetSyn components including hypertension and T2DM, to prevent CVD in this population.

This study had several limitations. Since it was cross-sectional, it is not possible to establish temporality of the observed relationships. Although our anonymous surveys of women who declined to participate showed no significant demographic differences from participants, the study used a non-probability sample that may have introduced selection bias. Consequently, descriptive population estimates may be less accurate than findings from research using a probability sample. In addition, while the six slums where the study was carried out were randomly selected from a sampling frame of 63 communities officially designated as ‘notified’ slums by the Karnataka Slum Development Board, and the sample size was calculated with 99% confidence level, and a 5% margin of error with a population estimate of 39,029 slum-dwellers [38], the use of a non-probability sample may still limit generalizability. Random selection of a sample was not possible in this case because slum households do not have addresses and slum residents are highly mobile, with households changing location frequently based on police harassment, crime, and opportunity, making enumeration required for a representative sampling frame almost impossible. In spite of these limitations, our finding that 41.5% of slum-dwelling women had Metsyn is consistent with the literature. While we were unable to find any South Indian studies examining Metsyn in slum-dwellers, we did identify a study in Udupi, a city in the same state of Karnataka, that reported a similar proportion (42.3%) of females with Metsyn [45] and the results from PURSE-HIS, a large population-based study with 8,080 participants that assessed Metsyn in adult South Indian populations also found a 38.3% prevalence of Metsyn among urban females [46, 47]. The similarity of the estimates give us confidence that our results are reasonably consistent with the literature. Some factors were self-reported so there is a possibility of recall and social acceptability bias. It is also possible the observed associations were subject to unmeasured or residual confounding. Finally, this study used HbA1c to assess average blood glucose in a population with elevated levels of iron deficiency anemia which has been associated with higher A1C concentrations, independent of glycemia [48]. Studies have suggested as much as an 8% elevation in mean HbA1c among anemic participants compared with non-anemic participants [49]. We assessed whether such a difference would alter the number of women meeting the HbA1c criteria, and found no difference in participant assignment in this study. In addition, higher education, income, religion, currently using smokeless tobacco, eating Western sweets, eating pickles, and consuming sodas, were not independently associated with MetSyn in the multivariable analysis in contrast to other studies. Our study may not have been adequately powered to show these relationships, or there may have been unmeasured confounding. With those weaknesses acknowledged, the study also had a number of important strengths. Biological and anthropometric data were collected in a clinical setting by trained medical personnel. The questionnaire was previously validated in similar Indian populations, adapted and pilot tested among slum-dwellers in Mysore, and administered by trained interviewers familiar with study communities. Finally, the study sample was highly homogenous for sociodemographic factors, and this may have lessened the potential risk for uncontrolled and residual confounding.

Results from this study suggest that slum-dwelling Indian women are at high-risk for MetSyn—a cluster of metabolic symptoms predictive of the prevalence and incidence of coronary heart disease, ischemic stroke, carotid artery disease, and diabetes. In countries like India, the relative simplicity of MetSyn criteria lend themselves to general clinical practice and prevention of heart disease and diabetes. Many of the participants in this study would benefit from lifestyle interventions to prevent or control MetSyn. There is strong evidence of an inverse association between cardiorespiratory fitness and MetSyn for women adopting a more physically active lifestyle [50]. The greatest opportunities lie in targeting this population with anti-hypertensive, antihyperlipidemic, and antidiabetic treatments all of which have been shown to significantly reduce prevalence, incidence, and sequelae of diabetes, CVD, and stroke.

There is also a pressing need for additional research to assess the prevalence of MetSyn in slum- dwelling populations across India. While our study suggests a substantially higher prevalence compared to other urban populations, there is a need to confirm these observations in other Indian settings. There is also an urgent public health need for interventions focused on particular components of MetSyn like hypertension, and education focused on changing unhealthy lifestyles that contribute to metabolic disease in this population.

## Supporting information

S1 Data Dataset used for this analysis is included as S1 Data. (XLS)

## References

[1] Nolan PB, Carrick-Ranson G, Stinear JW, Reading SA, Dalleck LC. Prevalence of metabolic syndrome and metabolic syndrome components in young adults: A pooled analysis. Prev Med Rep. 2017;7:211–5. doi: 10.1016/j.pmedr.2017.07.004 28794957 PMC5540707

[2] Ibrahim MS, Pang D, Randhawa G, Pappas Y. Risk models and scores for metabolic syndrome: systematic review protocol. BMJ Open. 2019;9(9):e027326. doi: 10.1136/bmjopen-2018-027326 31562141 PMC6773348

[3] Krishnamoorthy Y RS, Murali S, Rehman T, Sahoo J, Kar SS. Prevalence of metabolic syndrome among adult population in India: A systematic review and meta-analysis. PLoS One. 2020;15(10):e0240971. doi: 10.1371/journal.pone.0240971 33075086 PMC7571716

[4] Johnson LW, Weinstock RS. The metabolic syndrome: concepts and controversy. Mayo Clin Proc. 2006;81(12):1615–20. doi: 10.4065/81.12.1615 17165640

[5] Nishizawa H, Shimomura I. Population Approaches Targeting Metabolic Syndrome Focusing on Japanese Trials. Nutrients. 2019;11(6):1430. doi: 10.3390/nu11061430 31242621 PMC6627423

[6] Jiang X, Yang Z, Wang S, Deng S. "Big Data" Approaches for Prevention of the Metabolic Syndrome. Front Genet. 2022;13:810152. doi: 10.3389/fgene.2022.810152 35571045 PMC9095427

[7] Thor SM, Yau JW, Ramadas A. Nutritional and lifestyle intervention strategies for metabolic syndrome in Southeast Asia: A scoping review of recent evidence. PLoS One. 2021;16(9):e0257433. doi: 10.1371/journal.pone.0257433 34520483 PMC8439470

[8] Sayon-Orea C, Razquin C, Bullo M, Corella D, Fito M, Romaguera D, et al. Effect of a Nutritional and Behavioral Intervention on Energy-Reduced Mediterranean Diet Adherence Among Patients With Metabolic Syndrome: Interim Analysis of the PREDIMED-Plus Randomized Clinical Trial. JAMA. 2019;322(15):1486–99. doi: 10.1001/jama.2019.14630 31613346 PMC6802271

[9] Nakao YM, Miyamoto Y, Ueshima K, Nakao K, Nakai M, Nishimura K, et al. Effectiveness of nationwide screening and lifestyle intervention for abdominal obesity and cardiometabolic risks in Japan: The metabolic syndrome and comprehensive lifestyle intervention study on nationwide database in Japan (MetS ACTION-J study). PLoS One. 2018;13(1):e0190862. doi: 10.1371/journal.pone.0190862 29315322 PMC5760033

[10] Alfawaz HA, Wani K, Alnaami AM, Al-Saleh Y, Aljohani NJ, Al-Attas OS, et al. Effects of Different Dietary and Lifestyle Modification Therapies on Metabolic Syndrome in Prediabetic Arab Patients: A 12-Month Longitudinal Study. Nutrients. 2018;10(3):383. doi: 10.3390/nu10030383 29558423 PMC5872801

[11] Boudreau DM, Malone DC, Raebel MA, Fishman PA, Nichols GA, Feldstein AC. Health care utilization and costs by metabolic syndrome risk factors. Metab Syndr Relat Disord. 2009;7(4):305–14. doi: 10.1089/met.2008.0070 19558267

[12] Curtis LH, Hammill BG, Bethel MA, Anstrom KJ, Gottdiener JS, Schulman KA. Costs of the Metabolic Syndrome in Elderly Individuals. Diabetes Care. 2007;30(10):2553–8. 17623825 10.2337/dc07-0460

[13] Gupta I, Ranjan A. Public expenditure on Non-Communicable Diseases & Injuries in India: A budget-based analysis. PLoS One. 2019;14(9):e0222086. 31513623 10.1371/journal.pone.0222086 PMC6742225

[14] Bloom DE, Cafiero ET, McGovern ME, Prettner K, Stanciole A, Weiss J, et al. The Economic Impact of Non-Communicable Disease in China and India: Estimates, Projections, and Comparisons. Institute for the Study of Labor (IZA), IZA Discussion Paper Series, Discussion Paper No 7563. 2013; Found at: https://docs.iza.org/dp7563.pdf. Accessed on Jan 25, 2022.

[15] Khan Y, Lalchandani A, Gupta AC, Khadanga S, Kumar S. Prevalence of metabolic syndrome crossing 40% in Northern India: Time to act fast before it runs out of proportions. J Family Med Prim Care. 2018;7(1):118–23. doi: 10.4103/jfmpc.jfmpc_10_17 29915744 PMC5958552

[16] Sharma R, Sharma R, Kumar A. Metabolic Syndrome: Prevalence (IDF & NCEP-ATP III) in Udhampur, Jammu City. Int J Pharm Sci Res 2019;10(3):1420–5.

[17] Ravikiran M, Bhansali A, Ravikumar P, Bhansali S, Dutta P, Thakur JS, et al. Prevalence and risk factors of metabolic syndrome among Asian Indians: a community survey. Diabetes Res Clin Pract. 2010;89(2):181–8. doi: 10.1016/j.diabres.2010.03.010 20381187

[18] Kapil U, Khandelwal R, Ramakrishnan L, Khenduja P, Gupta A, Sareen N, et al. Prevalence of metabolic syndrome and associated risk factors among geriatric population living in a high altitude region of rural Uttarakhand, India. J Family Med Prim Care. 2018;7(4):709–16. doi: 10.4103/jfmpc.jfmpc_261_17 30234042 PMC6131997

[19] Sawant A, Mankeshwar R, Shah S, Raghavan R, Dhongde G, Raje H, et al. Prevalence of metabolic syndrome in urban India. Cholesterol. 2011;2011:920983. doi: 10.1155/2011/920983 21687582 PMC3114375

[20] Madan JG, Narsaria AM. Prevalence of metabolic syndrome in Mumbai City, India. J Obes Metab Res. 2016;3(1):16–22.

[21] Kamble P, Deshmukh PR, Garg N. Metabolic syndrome in adult population of rural Wardha, central India. Indian J Med Res. 2010;132(6):701–5. 21245618 PMC3102458

[22] Barik A, Das K, Chowdhury A, Rai RK. Metabolic syndrome among rural Indian adults. Clin Nutr ESPEN. 2018;23:129–35. doi: 10.1016/j.clnesp.2017.11.002 29460788

[23] Harikrishnan S, Sarma S, Sanjay G, Jeemon P, Krishnan MN, Venugopal K, et al. Prevalence of metabolic syndrome and its risk factors in Kerala, South India: Analysis of a community based cross-sectional study. PLoS One. 2018;13(3):e0192372. doi: 10.1371/journal.pone.0192372 29584725 PMC5870937

[24] Venugopal V, Dongre AR, Saravanan S. Prevalence and Determinants of Metabolic Syndrome among the Rural Adult Population of Puducherry. Indian J Community Med. 2019;44(1):21–5. doi: 10.4103/ijcm.IJCM_132_18 30983708 PMC6437807

[25] Sarkar P, Mahadeva SK, Raghunath H, Upadhya S, Hamsa M. Metabolic syndrome and its components among population of Holalu village, Karnataka. Int J Med Sci Public Health. 2015;5(5):860–5.

[26] Prasad DS, Kabir Z, Dash AK, Das BC. Prevalence and risk factors for metabolic syndrome in Asian Indians: A community study from urban Eastern India. J Cardiovasc Dis Res. 2012;3(3):204–11. doi: 10.4103/0975-3583.98895 22923938 PMC3425027

[27] Zafar KS, Pious T, Singh PS, Gautam RK, Yadav SK, Singh P, et al. Prevalence of metabolic syndrome in a rural population- a cross sectional study from Western Uttar Pradesh, India. Int J Res Med Sci. 2017;5(5):2223–8.

[28] Government of India Office of the Registrar General $ Census Commissioner. Population Finder 2011. Found at: https://censusindiagovin/censuswebsite/data/population-finder. Accessed on Jan 25, 2022.

[29] Lavanya KM, Thomas V, Muralidhar, Rao N. Metabolic Syndrome (Ms) among Adults in Urban Slums–A Cross Sectional Study in Hyderabad, Andhra Pradesh, India. J Community Med Health Educ 2012;2:192.

[30] Sinha S, Misra P, Kant S, Krishnan A, Nongkynrih B, Vikram NK. Prevalence of metabolic syndrome and its selected determinants among urban adult women in South Delhi, India. Postgrad Medical J. 2013;89(1048):68–72. doi: 10.1136/postgradmedj-2012-130851 23112218

[31] Prabhakaran D, Chaturvedi V, Shah P, Manhapra A, Jeemon P, Shah B, et al. Differences in the prevalence of metabolic syndrome in urban and rural India: a problem of urbanization. Chronic Illn. 2007;3(1):8–19. doi: 10.1177/1742395307079197 18072694

[32] Acharyya T, Kaur P, Murhekar MV. Prevalence of behavioral risk factors, overweight and hypertension in the urban slums of North 24 Parganas District, West Bengal, India, 2010. Indian J Public Health. 2014;58(3):195–8. doi: 10.4103/0019-557X.138632 25116826

[33] Shivaraj BM, Kiran BS, Ranganath, TS. Prevalence of hypertension and diabetes mellitus at selected urban slums in bangalore: a cross sectional study. J Evol Med Dent Sci. 2015;4(58):10077.

[34] Mukherjee K. Study on tobacco consumption patterns and its determinants in an urban slum in New Mumbai. Int J Epidemiol Res. 2015;2:164–71.

[35] Bollyky TJ, Templin T, Cohen M, Dieleman JL. Lower-Income Countries That Face The Most Rapid Shift In Noncommunicable Disease Burden Are Also The Least Prepared. Health Affairs. 2017;36(11):1863–2031. 29137514 10.1377/hlthaff.2017.0708 PMC7705176

[36] Sharma SK, Vishwakarma D, Puri P. Gender disparities in the burden of non- communicable diseases in India: Evidence from the cross-sectional study. Clin Epidemiol Glob Health. 2020;8(2):544–9.

[37] Arokiasamy P. India’s escalating burden of non-communicable diseases. Lancet Glob Health. 2018;6(12):e1262–e3. doi: 10.1016/S2214-109X(18)30448-0 30292427

[38] Government of India Office of the Registrar General $ Census Commissioner. Mysore City Population. 2011. Found at: https://wwwcensus2011coin/census/city/452-mysorehtml Accessed on Jan 25, 2022.

[39] Nair M, Ali MK, Ajay VS, Shivashankar R, Mohan V, Pradeepa R, et al. CARRS Surveillance study: design and methods to assess burdens from multiple perspectives. BMC Public Health. 2012;12:701. doi: 10.1186/1471-2458-12-701 22928740 PMC3491014

[40] Beaton DE, Bombardier C, Guillemin F, Ferraz MB. Guidelines for the process of cross-cultural adaptation of self-report measures. Spine (Phila Pa 1976). 2000;25(24):3186–91. doi: 10.1097/00007632-200012150-00014 11124735

[41] Alberti KG, Eckel RH, Grundy SM, Zimmet PZ, Cleeman JI, Donato KA, et al. Harmonizing the metabolic syndrome: a joint interim statement of the International Diabetes Federation Task Force on Epidemiology and Prevention; National Heart, Lung, and Blood Institute; American Heart Association; World Heart Federation; International Atherosclerosis Society; and International Association for the Study of Obesity. Circulation. 2009;120(16):1640–5. doi: 10.1161/CIRCULATIONAHA.109.192644 19805654

[42] American Diabetes Association. Standards of medical care in diabetes—2014. Diabetes Care. 2014;37 Suppl 1:S14–80. doi: 10.2337/dc14-S014 24357209

[43] Montgomery D C, Peck E A, Vining GG. Introduction to linear regression analysis. 3rd edition. New York, NY: Wiley; 2001. p. 194.

[44] Blazer DG, Hybels CF, Fillenbaum GG. Metabolic syndrome predicts mobility decline in a community-based sample of older adults. J Am Geriatr Soc. 2006;54(3):502–6. doi: 10.1111/j.1532-5415.2005.00607.x 16551320

[45] Pai NN, Meenakshi G. Metabolic syndrome risk assessment among adults in Udupi District, Karnataka. Clin Epidemiol Glob Health. 2020;8(1):142–8.

[46] Joshi C, Thanikachalam M, Bermudez OI, Chui KKH. Disparities in prevalence of metabolic syndrome: A cross-sectional analysis of Indian adults. Eur J Public Health. 2020;30(Supp 5):v932.

[47] Thanikachalam S, Harivanzan V, Mahadevan MV, Murthy JS, Anbarasi C, Saravanababu CS, et al. Population Study of Urban, Rural, and Semiurban Regions for the Detection of Endovascular Disease and Prevalence of Risk Factors and Holistic Intervention Study: Rationale, Study Design, and Baseline Characteristics of PURSE-HIS. Glob Heart. 2015;10(4):281–9. doi: 10.1016/j.gheart.2014.11.002 26014656

[48] Sahay M, Kalra S, Tiwaskar M, Ghosh S, Badani R, Bantwal G, et al. Indian College of Physicians Position Statement on Anemia in Metabolic Syndrome. J Assoc Physicians India. 2017;65(6):60–73. 28782315

[49] Adeoye S, Abraham S, Erlikh I, Sarfraz S, Borda T, Yeun L. Anaemia and Haemoglobin A1c level: Is there a case for redefining reference ranges and therapeutic goals? BJMP. 2014;7(1):a706.

[50] Kim S. Association between Cardiorespiratory Fitness and Metabolic Syndrome in Korean Older Adults. Int J Environ Res Public Health. 2022;19(19):3671. doi: 10.3390/ijerph19063671 35329357 PMC8950222

